# The effect of ultrasound-guided intercostal nerve block on postoperative analgesia in thoracoscopic surgery: a randomized, double-blinded, clinical trial

**DOI:** 10.1186/s13019-023-02210-8

**Published:** 2023-04-11

**Authors:** Shuo Li, Jinteng Feng, Kun Fan, Xiaoe Fan, Shaoning Cao, Guangjian Zhang

**Affiliations:** Department of Thoracic Surgery, the First Affiliated Hospital of Xi’an Jiaotong University, 277 Yanta West Road, Xi’an, Shaanxi China

**Keywords:** Postoperative pain, Ultrasound guidance, Intercostal nerve block, Thoracoscopic surgery

## Abstract

**Background:**

Intercostal nerve block (ICNB) is a very effective analgesic method. We aimed to explore the effect of preemptive analgesia with ultrasound-guided intercostal nerve block on postoperative analgesia in thoracoscopic surgery.

**Methods:**

126 patients, aged 18–70 years, with American Society of Anesthesiologists (ASA) physical status I-II and scheduled for thoracoscopic pulmonary resection were enrolled in this study. 119 patients were left for final analysis. Patients were randomly allocated to group ICNB and group CONTROL. Patients in CONTROL group were administered sufentanil with patient-controlled analgesia device after operation In group ICNB, patients received ropivacaine ICNB prior to surgery and patient-controlled analgesia device after operation. The primary outcome is visual analog scale pain score (VAS) at rest at 0,4, 8,16,24,48,72 and 168 h postoperatively and they were compared. Surgical outcomes and rescue analgesia requirement were also recorded.

**Results:**

VAS scores were statistically significantly lower for ICNB group compared to control group at 0, 4, 8, 16, 24 and 48 h postoperatively. The duration of insertion of chest tube in ICBN group was shorter than that in control group, and the difference was statistically significant (4.69 ± 2.14 vs. 5.67 ± 2.86, *P* = 0.036). The postoperative hospital stay, incidence of nausea and vomiting and postoperative pulmonary infection rate in ICBN group were all lower than those in the control group, but there were no statistical differences. The frequency of rescue analgesia during 48 postoperative hours was different between the two groups (ICNB vs. Control; 9.83% vs. 31.03%, *P* = 0.004).

**Conclusions:**

For patients undergoing thoracoscopic surgery, ultrasound-guided ICNB is simple, safe, and effective for providing acute postoperative pain management during the early postoperative stage.

**Trial registration:**

Chinese clinical trials: chictr.org.cn, ChiCTR1900021017. Registred on 25/01/2019.

**Supplementary Information:**

The online version contains supplementary material available at 10.1186/s13019-023-02210-8.

## Background

Thoracoscopy has been widely used in thoracic surgery, with advantages including less systemic trauma, shorter length of hospital stay and fewer postoperative complications [1]. Moderate-to-severe pain usually occurred after thoracoscopic surgery and increased patients’ suffering and made patients unable to breathe normally and have an infective cough, which will increase respiratory complications. Meanwhile, acute pain may become chronic pain, reducing the quality of life of patients after surgery [2, 3]. Multimodal analgesia is accepted perioperative [4], intercostal nerve block (ICNB) which use ropivacaine is a kind of methods of multimodal analgesia. ICNB has the advantages of easy to operate and blocking the local intercostal nerve pain directly. We designed a randomized, double-blinded, clinical trial to explore the effect of ultrasound-guided intercostal nerve block on postoperative analgesia in thoracoscopic surgery. In the current study we tested the hypothesis that ICNB relieves postoperative pain without increasing side effects.

## Patients and methods

### Patients

This was a single center with balanced randomization, double-blind, parallel-group study. This study was approved by Ethics Committee of the First Affiliated Hospital of Xi ‘an Jiaotong University (NO.2018G-22) and registered in Chinese Clinical Trials Registry (Registration No: ChiCTR1900021017, Date of Registration, 25/01/2019). **The design and implementation of the study were conducted in accordance with the Helsinki Declaration.** Recruitment was performed between October 2019 and August 2020. Written informed consents were obtained from all patients.

Patients aged 18–70 years old with American Society of Anesthesiologists (ASA) physical status I-II and undergoing thoracoscopic pulmonary resection in our hospital were enrolled in the study. The exclusion criteria included allergy to local anesthesia, severe heart diseases, hepatic or renal insufficiency, a history of chronic pain or chronic opioid use and psychiatric disease.

Using a simple randomization procedure with computer-generated allocation, patients were allocated randomly to intercostal nerve block group (ICNB) and control group. The allocation sequence was concealed from the investigator enrolling and assessing participants by using sequentially-numbered, opaque, sealed, and stapled envelopes. All patients and the investigator who was responsible for follow-up postoperative were blinded to the randomization groups. In addition, before surgery we educated patients how to use the visual analog scale (VAS) to evaluate pain.

The primary outcome is visual analog scale pain score (VAS) at rest at 0,4, 8,16,24,48,72 and 168 h postoperatively. The surgical outcomes including length of postoperative stay, duration of insertion of chest tube, incidence of nausea and vomiting and postoperative pulmonary infection were recorded and the rescue analgesia requirement in the two groups was also recorded.

## Methods

### General anesthesia and surgical technique

All patients received oral celecoxib 300 mg, twice a day in the first three days before surgery. Patients were monitored using an electrocardiogram, pulse oximetry and invasive blood pressure during the surgery. General anesthetic induction was conducted with sufentanil 0.5 µg·kg^-1^, propofol 1.5-2.0 mg·kg^-1^ and rocuronium µ0.8 mg·kg-1. Anesthesia was maintained with and intravenous infusion of dexmedetomidine at 0.5 µg·kg^-1^·h^-1^ and remifentanil at0.1-0.2 µg·kg^-1^·min^-1^.

The patient was placed in the lateral decubitus position, and the same anesthesiologist performed ultrasound-guided intercostal nerve block with ropivacaine prior to skin incision. Standard three-hole thoracoscopic surgery was performed. The observation hole was the 7th or 8th intercostal in the midaxillary line, with a length of about 1 cm; the main operating hole was the 4th or 5th intercostal in the anterior axillary line, with a length of about 3 cm; and the minor operating hole was the 8th or 9th intercostal in the subscapular angle line. After the operation, a thoracic drainage tube was placed in the observation hole.

### Ultrasound-guided intercostal nerve block [5]

The patient was in the lateral decubitus position. Unilateral ICNB was performed at the levels of T3-T10. A linear M-Turbo ultrasound probe (FUJIFILM SonoSite, Washington, America) was placed in a longitudinal orientation to identify the rib, internal intercostal muscles, innermost intercostal muscles and pleura. An 18-gauge, 10-cm needle (TUOREN, Henan, China) was inserted to target the inferior margin of the rib at the proximal side of intercostal nerve in an inplane approach. After negative aspiration, the anesthesiologist injected 4 ml of 0.375% ropivacaine into the intercostal spaces where three incisions located and 3 ml ropivacaine for other levels. When pleural displacement was observed in all intercostal spaces, ICNB was considered successful.

### Postoperative analgesia protocol and rescue analgesic

After surgery all patients were connected to the PCA device (Rehn Medical, Nantong, Jiangsu, China). The PCA device consisted of 1.5 µg·ml^− 1^ sufentanil and was programmed as follow: 2 ml·h^− 1^ background rate, 1ml bolus- doses and 15 min-lockout intervals. Upon arrival at ward (0 h after surgery), patients were requested to evaluate pain at rest using visual analog scale (VAS: 0 = no pain, 10 = worst pain imaginable). If VAS score was > 3 at rest in the ward, Parecoxib Sodium for injection 40 mg were given as rescue analgesia.

### Statistical analysis

The data were analyzed based on the intention to treat method. Statistical analyses were performed with SPSS 17.0 (SPSS Inc., Chicago, IL, USA). The values were expressed as the mean ± standard deviation (SD). An unpaired Student’s t test was used to test continuous variables. The Chi-square test was used to compare categorical variables. *p* values of < 0.05 were considered to indicate statistical significance.

Sample size calculation was based on a pilot study that showed the difference of VAS scores were 1.6 between the two groups within 24 h postoperative. Choosing a difference of 1 score as the minimum desired difference between the groups, 37 patients per group were required to achieve a significance level of 0.05 with a power of 95%. Combined with the number of operations in our department and to compensate for dropouts, we planned to recruit 67 patients in each group.

## Results

**Fig. 1:**
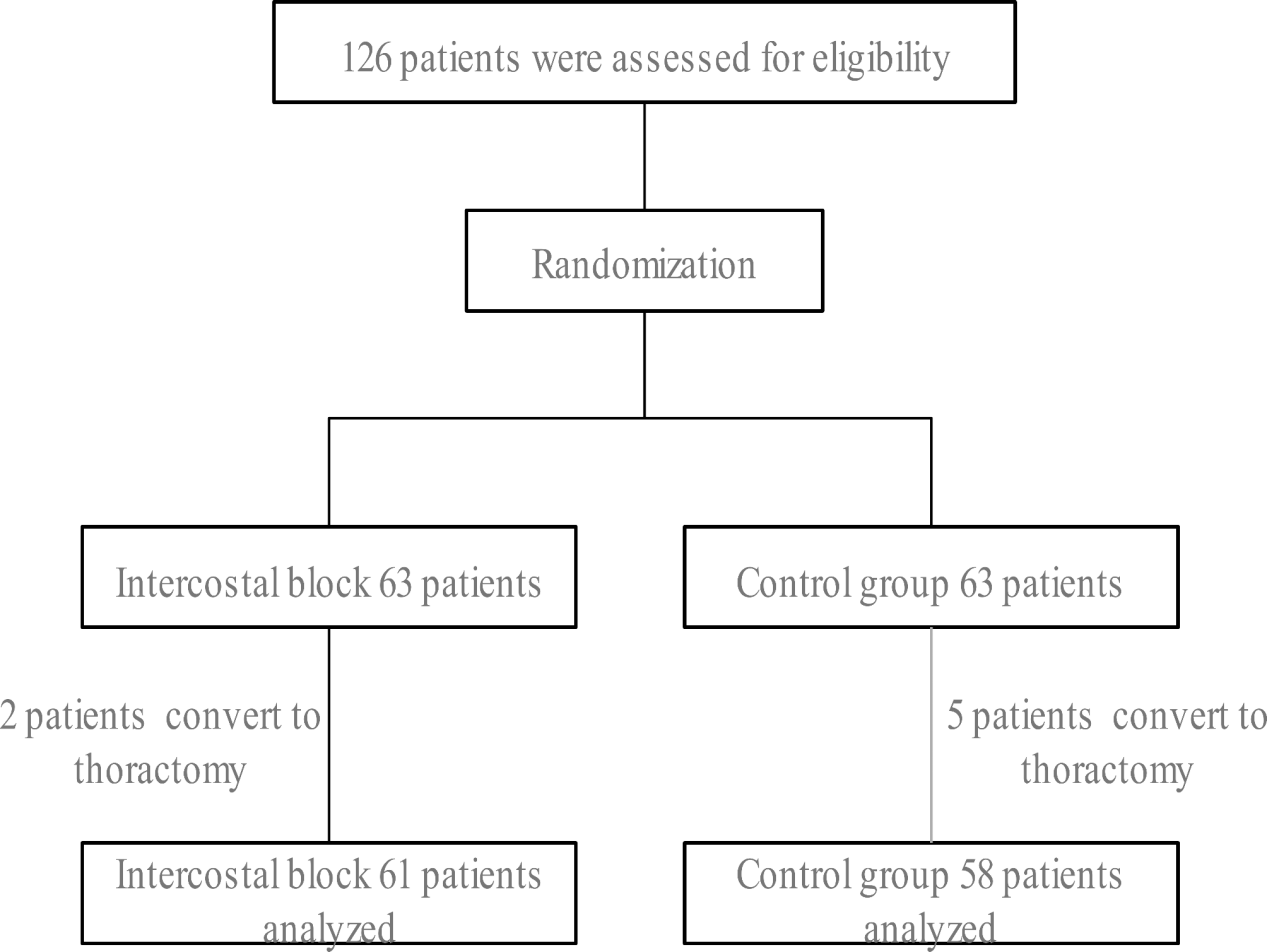
Schematic illustration of the study population

126 patients were assessed for eligibility. 7 patients were excluded from final analysis because of converting to thoracotomy. Finally, 119 patients completed the study (Fig. 1). The baseline demographics and perioperative variables were similar in the two groups (Table 1). All patients underwent pulmonary resection thoracoscopic via a thoracoscopic procedure.

**Fig. 2:**
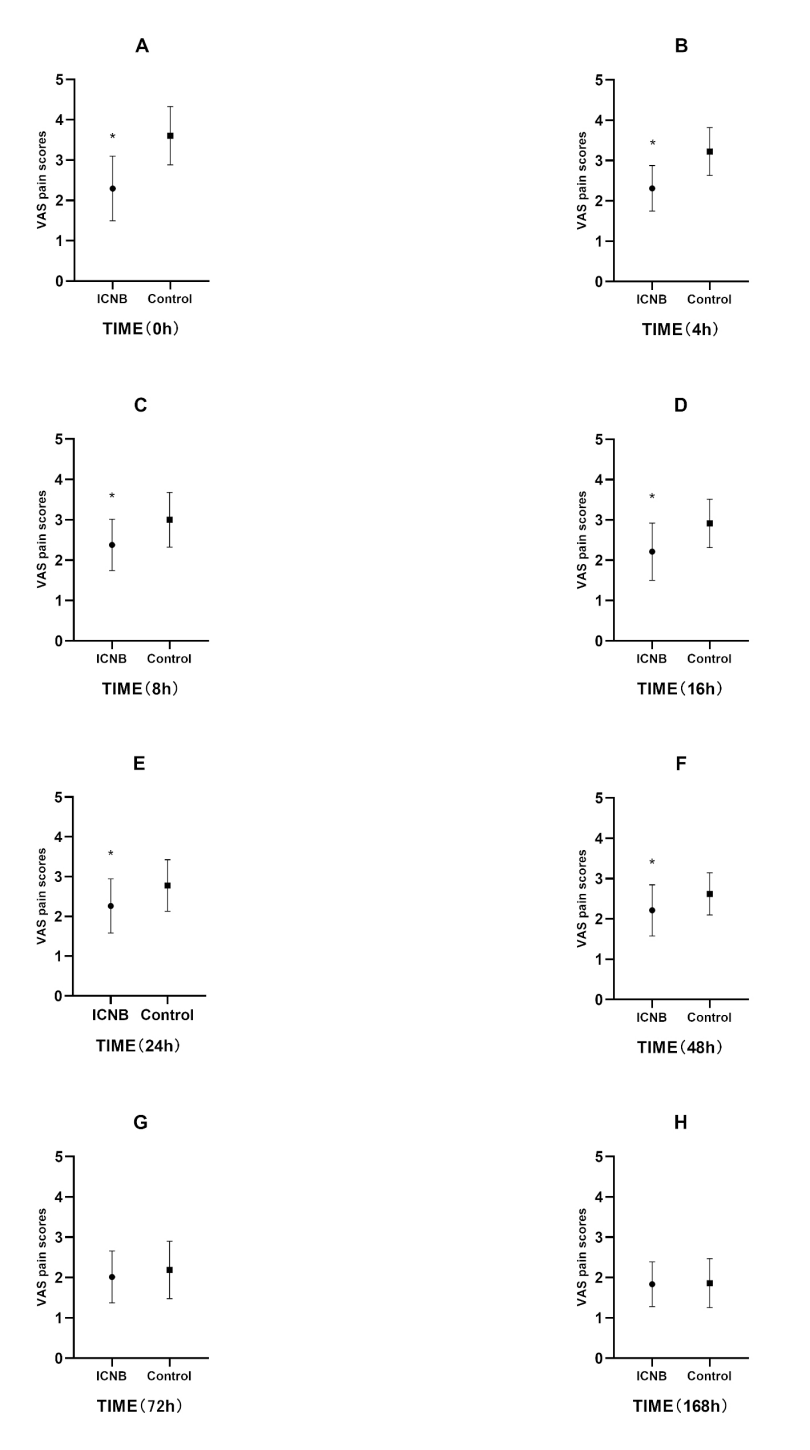
Comparison of visual analog scale (VAS) pain scores with respect to time. * *P* < 0.001

**Fig. 3:**
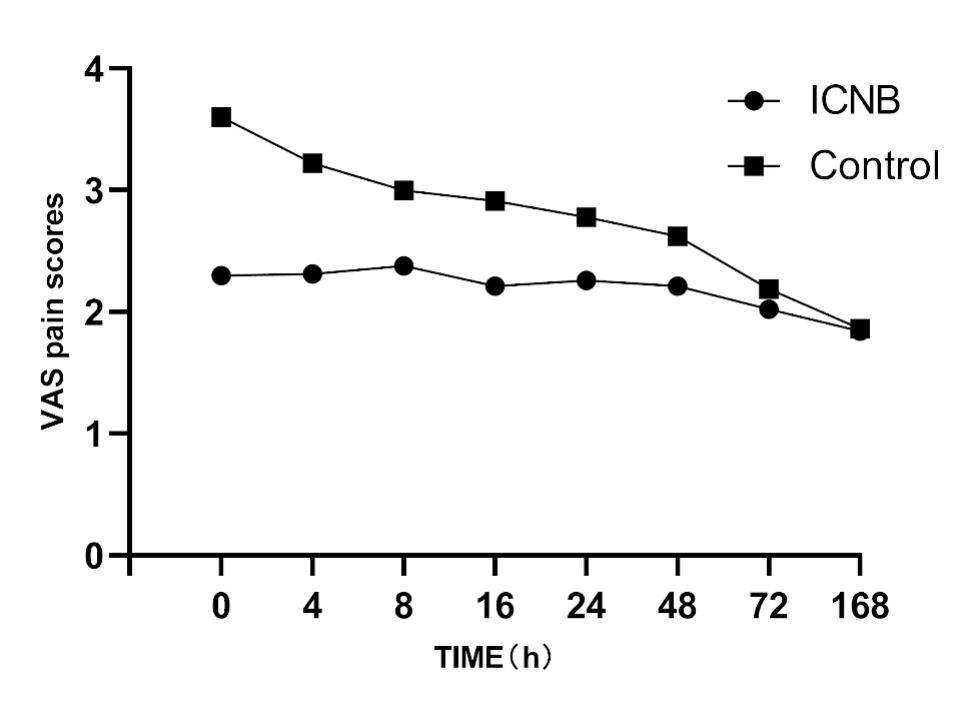
Visual analog scale (VAS) pain scores with respect to time

AS indicated in Fig. 2; Table 2, VAS scores were statistically significantly lower for ICNB group compared to control group at 0, 4, 8, 16, 24 and 48 h postoperatively. The VAS scores of ICNB group were lower than the control group at each time point and tended to be the same after 48 h postoperatively (Fig. 3).

The duration of insertion of chest tube in ICBN group was shorter than that in control group, and the difference was statistically significant (4.69 ± 2.14 vs. 5.67 ± 2.86, *P* = 0.036). The postoperative hospital stay, incidence of nausea and vomiting and postoperative pulmonary infection rate in ICBN group were all lower than those in the control group, but there were no statistical differences (Table 3).

**Table 1:** Demographic and perioperative characteristics

	ICNB	CONTROL	*t/χ* ^*2*^	*P*
Sex F M Age(yr) Duration of surgery(min) Type of VATS Lobectomy Segmentectomy			0.006	0.936
	32	30		
	29	28		
	57.84 ± 9.21	56.91 ± 10.90	0.499	0.619
	111.84 ± 52.49	126.74 ± 61.62	-1.423	0.157
			0.492	0.782
	5	7		
	11	10		
Wedge resection	45	41		
Weight(kg)	66.39 ± 13.01	62.62 ± 10.59	2.304	1.725
Height(cm) Body Mass Index(kg/m^2^)	166.46 ± 8.48 23.67 ± 83.26	164.64 ± 7.99 23.22 ± 4.14	0.138 0.749	1.204 0.661
ASA class I II	25 36	23 35	0.022	0.883

**Table 2:** Visual analog scale (VAS) pain scores with respect to time

TIME (h)	ICNB	CONTROL	*t*	*P*
0 4 8 16 24 48 72 168	2.30 ± 0.10	3.60 ± 0.09	-9.319	< 0.001
	2.31 ± 0.07	3.22 ± 0.07	-8.601	< 0.001
	2.38 ± 0.08	3.00 ± 0.89	-5.179	< 0.001
	2.21 ± 0.09	2.91 ± 0.07	-5.798	< 0.001
	2.26 ± 0.08 2.21 ± 0.08	2.78 ± 0.08	-4.210	< 0.001
		2.62 ± 0.06	-3.825	< 0.001
	2.02 ± 0.08	2.19 ± 0.09	-1.392	0.167
	1.84 ± 0.07	1.86 ± 0.08	-0.245	0.807

**Table 3:** Surgical outcomes

Variables	ICNB(n = 61)		CONTROL(n = 58)	*t/χ* ^*2*^	*P*
length of postoperative stay(d) duration of insertion of chest tube(d) incidence of nausea and vomiting (%) postoperative pulmonary infection (%)	7.18 ± 2.46	7.88 ± 2.82		-1.439	0.153
	4.69 ± 2.14	5.67 ± 2.86		-2.215	0.036
	2(3.27%) 3(4.91%)	5(8.62%) 6(10.34%)		0.719 0.596	0.396 0.440

**Table 4:** Rescue analgesia requirement

Variables	ICNB(n = 61)		CONTROL(n = 58)	*χ* ^*2*^	*P*
0-4 h (%) 0-8 h (%) 0-24 h (%) 0-48 h (%)	3(4.91%)	8(13.79%)		2.791	0.095
	5(8.19%)	12(20.68%)		3.790	0.052
	6(9.83%) 6(9.83%)	15(25.86%) 18(31.03%)		5.254 8.298	0.022 0.004

The rescue analgesia requirement in the two groups was shown in Table 4. The frequency of rescue analgesia during 48 postoperative hours was different between the two groups (ICNB vs. Control; 9.83% vs. 31.03%, *P* = 0.004).

## Discussion

In this study, we explored the effect of ultrasound-guided intercostal nerve block on postoperative analgesia in thoracoscopic surgery. Whatever in ICNB group or control group, none of patients had severe pain (VAS>6). The results indicate that multimodal analgesia [6] can effectively prevent the occurrence of severe pain. In this study, multimodal analgesia has achieved good effects, and this mode has been more and more recognized and accepted [7, 8].

In this study, ICNB was used as an advance analgesic strategy to relieve thoracoscopic postoperative pain. Our results showed that ICNB could alleviate pain in the early stage after thoracoscopic surgery, reduce the VAS pain scores within 48 h after the surgery. With the multimodal analgesia used in the control group, about 30% of patients still needed rescue analgesia. In ICNB group, the incidence of rescue analgesia was reduced to 9.8%. Severe acute pain after thoracoscopic surgery is mainly due to the fracture or retraction of ribs and injury of intercostal nerves. ICNB can directly interrupts the flow of afferent pain signals through intercostal nerve and reduce pain intensity [9, 10]. The main side effect is pneumothorax with occurrence rate around 1%. Ultrasound guidance improves the method safety, and even if pneumothorax is performed before thoracoscopic surgery, it can be well managed.

Regional anesthesia techniques which used in thoracoscopic surgery include thoracic epidural analgesia (TEA), thoracic paravertebral block (PVB), erector spinae plane block (ESPB) and ICNB. TEA was once considered as the gold standard for post-thoracotomy pain management, but it is associated with high potential risks of dural puncture, nerve lesions, peidural hematoma and hypotension [10]. PVB is not in regular use because of technical challenge and potential risks [11, 12]. Similar to PVB, ESPB also requires certain technology [13]. Ultrasound-guided ICNB is simple, safe and effective regimen for pain control after thoracoscopic surgery and can be used as a valid option especially when anesthesiologists have little experience in TEA and PVB or when PVB is contraindicated of failed [14]. Similar to previous study [15], the duration of ICNB is about 48 h, and continuous intercostal nerve injection could prolong the duration of action [16].

The duration of insertion of chest tube and postoperative pulmonary infection rate were lower than those in the control group. The reason was that the patients could act the respiratory function better under the good analgesic effect.

Good postoperative pain management can provide conditions for patients to perform respiratory function training, so our study showed the less duration of insertion of chest tube and less incidence of postoperative pulmonary infection. Also, it could decrease the incidence of nausea and vomiting which caused by opioid analgesics [17]. The length of postoperative stay was also less in ICNB group than in the control group and we believe the ICNB is the more cost-effective strategy.

Our study has some limitations. First, we evaluated the VAS for pain at rest but not while coughing. The VAS is generally higher when coughing than at rest. A more comprehensive assessment of the postoperative pain might provide more useful findings. Second, we chose the VAS scores as the analysis index, The VAS score is a subjective indicator rather than an objective indicator. We should combine some objective indicators to evaluate in the further study. Third, this study may strongly depend on the anesthesiologist’s or surgeon’s experience. Fourth, the study failed to count opioid use. Thus, more objective evaluation indicators can be sought in subsequent studies, and pain management can be individualized for patients.

## Conclusions

In summary, for patients undergoing thoracoscopic surgery, ultrasound-guided ICNB is simple, safe, and effective for providing acute postoperative pain management during the early postoperative stage.

## Electronic supplementary material

Below is the link to the electronic supplementary material.

**Additional File Figure 1**: Ethical review

**Additional File 1**: Statement

**Additional File 2**: Trial protocol V2.1

## Data Availability

The datasets generated and analysed during the current study are not publicly available due protect the participants’ identity but are available from the corresponding author on reasonable request.

## Abbreviations

ICNB: Intercostal nerve block
TEA: Thoracic epidural analgesia
PVB: Paravertebral block
VAS: Visual analog scale
SD: Standard deviation
PCA: Patient controlled analgesia
SPSS: Statistical package for the social sciences
ESPB: Erector spinae plane block
