# Supplementary figures and images for: The effect of ultrasound-guided intercostal nerve block on postoperative analgesia in thoracoscopic surgery: a randomized, double-blinded, clinical trial
Source: J Cardiothorac Surg. 2023 Apr 11;18:128. doi: 10.1186/s13019-023-02210-8 (PMC10091630; doi:10.1186/s13019-023-02210-8)

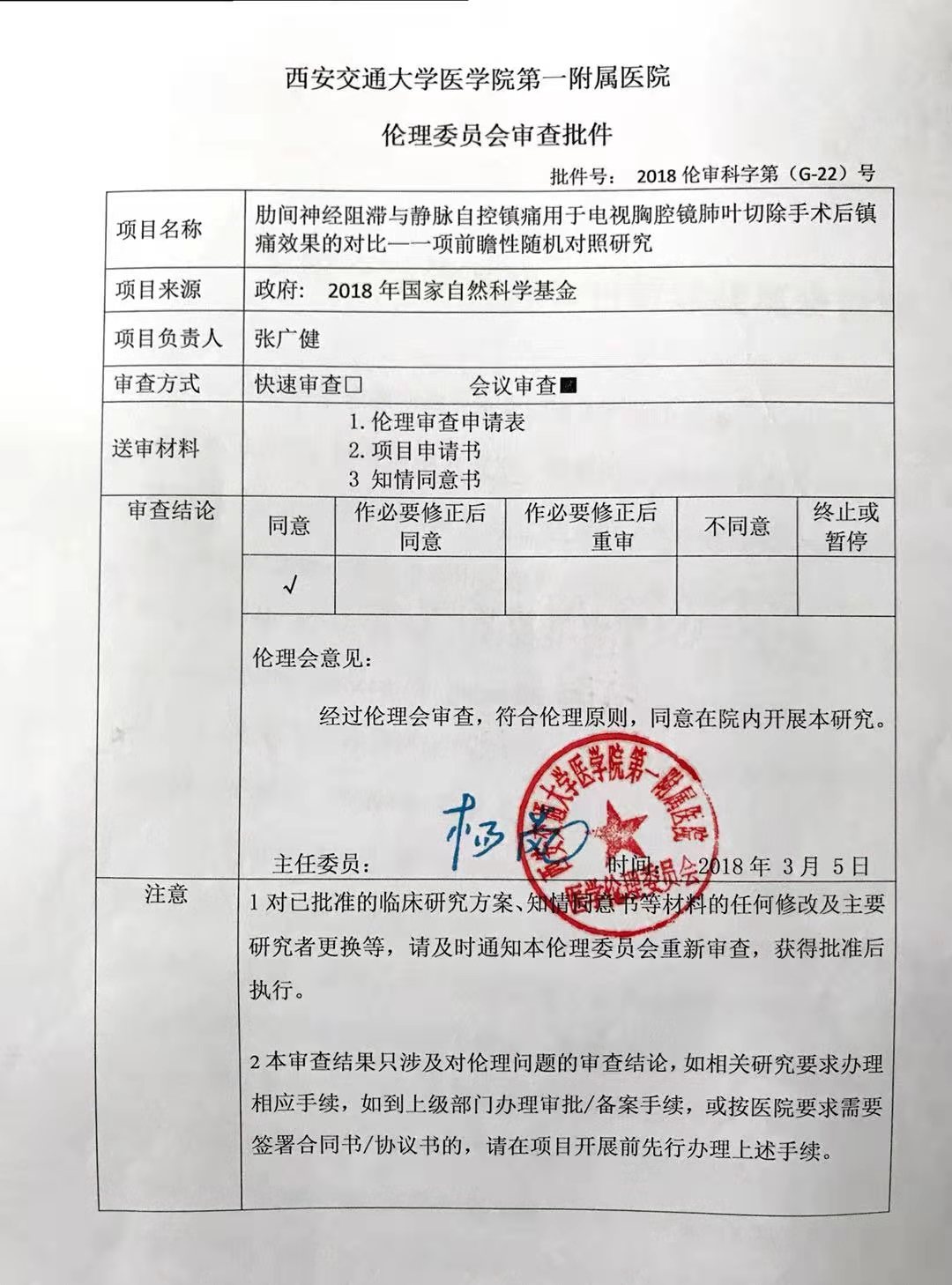

Supplement: Supplementary file 1 — Additional File Figure 1: Ethical review [file 13019_2023_2210_MOESM1_ESM.jpg]
