# Supplementary material for: The effect of ultrasound-guided intercostal nerve block on postoperative analgesia in thoracoscopic surgery: a randomized, double-blinded, clinical trial
Source: J Cardiothorac Surg. 2023 Apr 11;18:128. doi: 10.1186/s13019-023-02210-8 (PMC10091630; doi:10.1186/s13019-023-02210-8)
Supplement: Supplementary file 2 — Additional File 1: Statement [file 13019_2023_2210_MOESM2_ESM.docx]

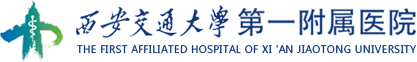


Professor Zhang Guangjian

Department of Thoracic Surgery

the First Affiliated Hospital of Xi 'an Jiaotong University

277 Yanta West Road, Xi 'an, Shaanxi China

TEL: 86-18991232613

Email: [michael8039@163.com](mailto:michael8039@163.com)

Journal of Cardiothoracic Surgery

May 05, 2022

**STATEMENT**

This study was approved by Ethics Committee of the First Affiliated Hospital of Xi 'an Jiaotong University (NO.2018G-22) and registered in Chinese Clinical Trials Registry (Registration No: ChiCTR1900021017). **The design and implementation of the study were conducted in accordance with the Helsinki Declaration.**
