# Supplementary material for: The effect of ultrasound-guided intercostal nerve block on postoperative analgesia in thoracoscopic surgery: a randomized, double-blinded, clinical trial
Source: J Cardiothorac Surg. 2023 Apr 11;18:128. doi: 10.1186/s13019-023-02210-8 (PMC10091630; doi:10.1186/s13019-023-02210-8)
Supplement: Supplementary file 3 — Additional File 2: Trial protocol V2.1 [file 13019_2023_2210_MOESM3_ESM.docx]

TRIAL PROTOCOL

**Title of Research Study**

The effect of ultrasound-guided intercostal nerve block on postoperative analgesia in thoracoscopic surgery: A randomized, double-blinded, clinical trial.

**Research purpose**

To explore the effect of ultrasound-guided intercostal nerve block on postoperative analgesia in thoracoscopic surgery.

**The research type**

Intervention study.

**Research design**

Randomized controlled trial.

**Key Interventions**

Patients were randomly assigned to receive thoracoscopic lobotomy and intercostal nerve block for analgesia or not.

**The research object**

**Clinical trial enrollment standard:** patients aged 18-70 years old with American Society of Anesthesiologists (ASA) physical status I-II and undergoing thoracoscopic pulmonary resection in our hospital were enrolled in the study.

**Clinical trial exclusion criteria:** allergy to local anesthesia, severe heart diseases, hepatic or renal insufficiency, a history of chronic pain or chronic opioid use and psychiatric disease.

**Elimination criteria for clinical trials:** transferred to thoracotomy during operation, two or more thoracic drainage tubes were indwailed for postoperative operation, severe postoperative complications, side effects of analgesia, special adverse events, etc., cannot be continued for observation or treatment, lost visitors.

**Sample size calculation**

Sample size calculation was based on a pilot study that showed the difference of VAS scores were 1.6 between the two groups within 24h postoperative. Choosing a difference of 1 score as the minimum desired difference between the groups, 37 patients per group were required to achieve a significance level of 0.05 with a power of 95%. Combined with the number of operations in our department and to compensate for dropouts, we planned to recruit 67 patients in each group.

**Primary endpoint**

The degree of pain at resting was assessed dynamically through the visual analogue scale (VAS) to 168 hours postoperatively.

**Statistical analysis**

Statistical analyses were performed with SPSS 17.0. The values were expressed as the mean ± standard deviation (SD). An unpaired Student’s t test was used to test continuous variables. The Chi-square test was used to compare categorical variables. *p* values of < 0.05 were considered to indicate statistical significance.

**Budget**

80000 yuan RMB

**Study process**

Patients admitted to the hospital during the trial period were selected as subjects according to the inclusion and exclusion criteria. All the subjects completed preoperative examinations. The trial administrators used simple randomization, using computer-generated random numbers, to randomly (double-blind) assign subjects to intercostal nerve block group and controlled analgesia group for analgesia in order of enrollment. During the experiment, neither the subjects nor the experimenter (including the surgeon) knew about the grouping. After the operation, the corresponding indexes were observed and recorded by the experimenter, which were counted by the trial manager.

**The research methods**

**Evaluation of analgesic effect:** The degree of pain was measured using Visual Analogue Scale (VAS), which is currently the most widely used pain intensity assessment method in clinical practice. A walking scale with a length of about 10cm was used. One side was marked with 10 scales, and the two ends were marked with "0" and "10" points respectively. 0 points meant painless, and 10 points meant the most intense pain that was unbearable. The patient faces the ungraduated side. According to the pain degree he or she feels, he or she places the cursor on the part that best represents the pain degree at that time. The doctor faces the graduated side and records the pain degree. VAS is a simple, effective, minimal-participation measure of pain intensity and has a high rate of successful response in the assessment of postoperative acute pain. The analog nature of the scale can accurately express the level of pain the patient is currently experiencing.

**Analgesic method:** All patients received oral celecoxib 300mg, twice a day in the first three days before surgery. Patients were monitored using an electrocardiogram, pulse oximetry and invasive blood pressure during the surgery. General anesthetic induction was conducted with sufentanil 0.5μg·kg^－1^, propofol 1.5-2.0mg·kg^－1^ and rocuronium 0.1-0.2μg·kg^－1^·min^－1^. Anesthesia was maintained with and intravenous infusion of dexmedetomidine at0.5μg·kg^－1^·h^－1^ and remifentanil at0.1-0.2μg·kg^－1^·min^－1^. The patient was in the lateral decubitus position, and the same anesthesiologist performed ultrasound-guided intercostal nerve block with ropivacaine prior to dermectomy. Standard three-hole thoracoscopic surgery was performed. The observation hole was the 7th or 8th intercostal in the midaxillary line, with a length of about 1cm; the main operating hole was the 4th or 5th intercostal in the anterior axillary line, with a length of about 3cm; and the minor operating hole was the 8th or 9th intercostal in the subscapular angle line. After the operation, a thoracic drainage tube was placed in the observation hole. After surgery all patients were connected to the PCA device (Rehn Medical, Nantong, Jiangsu, China). The PCA device consisted of 1.5 μg·ml^-1^ sufentanil and was programmed as follow: 2 ml·h^-1^ background rate, 1ml bolus- doses and 15 min-lockout intervals. Upon arrival at ward (0 h after surgery), patients were requested to evaluate pain at rest using visual analog scale (VAS: 0 = no pain, 10 = worst pain imaginable). If VAS score was >3 at rest in the ward, Parecoxib Sodium for injection 40mg were given as rescue analgesia.

**Obvervational index:** The baseline demographics and perioperative variables (gender, age, duration of surgery, type of VATS, weight, height, body mass index, ASA class). Visual analog scale (VAS) pain scores with respect to time (0, 4 ,8 ,16 ,24 ,48 ,72, 168h). Surgical outcomes (length of postoperative stay, duration of insertion of chest tube, incidence of nausea and vomiting, postoperative pulmonary infection) and rescue analgesia requirement.

**Standardized procedures for surgical operation**

Standard three-hole thoracoscopic surgery was performed. The observation hole was the 7th or 8th intercostal in the midaxillary line, with a length of about 1cm; the main operating hole was the 4th or 5th intercostal in the anterior axillary line, with a length of about 3cm; and the minor operating hole was the 8th or 9th intercostal in the subscapular angle line.

**Ultrasound-guided intercostal nerve block**

The patient was in supine position. Unilateral ICNB was performed at the levels of T4-T9. A linear M-Turbo ultrasound probe (FUJIFILM SonoSite, Washington, America) was placed in a longitudinal orientation to identify the rib, internal intercostal muscles, innermost intercostal muscles and pleura. An 18-gauge, 10-cm needle (TUOREN, Henan, China) was inserted to target the inferior margin of the rib in an inplane approach. After negative aspiration, the anesthesiologist injected 4 ml of 0.375% ropivacaine into the intercostal spaces where two incisions located and 3 ml ropivacaine for other levels. When pleural displacement was observed in all intercostal spaces, ICNB was considered successful.
